# Current status, challenges and prospects for pig production in Asia

**DOI:** 10.5713/ab.23.0303

**Published:** 2024-02-23

**Authors:** Lu Wang, Defa Li

**Affiliations:** 1 State Key Laboratory of Animal Nutrition, College of Animal Science and Technology, China Agricultural University, Beijing 100193, China

**Keywords:** Asia, Pig Production, Small-scale Pig Farms, Sustainability

## Abstract

Asia is not only the primary region for global pig production but also the largest consumer of pork worldwide. Although the pig production in Asia has made great progress in the past, it still is confronted with numerous challenges. These challenges include: inadequate land and feed resources, a substantial number of small-scale pig farms, escalating pressure to ensure environmental conservation, control of devastating infectious diseases, as well as coping with high temperatures and high humidity. To solve these problems, important investments of human and financial capital are required to promote large-scale production systems, exploit alternative feed resources, implement precision feeding, and focus on preventive medicine and vaccines as alternatives to antibiotics, improve pig breeding, and increase manure recycling. Implementation of these techniques and management practices will facilitate development of more environmentally-friendly and economically sustainable pig production systems in Asia, ultimately providing consumers with healthy pork products around the world.

## INTRODUCTION

Asia is not only the primary region for global pig production but also the largest consumer of pork worldwide. Pig production plays a crucial role in Asian countries, both as a source of food and as a contributor to the economy. Pork is an important source of animal protein for humans and accounts for more than 30% of total global meat production annually [1]. As demand for animal products increases, pork will continue to be one of the major sources of protein in Asia and pig production will continue to be one of the major farm animals in the livestock subsector [2]. In addition, the pig production is an important component of agricultural economies and trade. This industry is an effective means for farmers to increase their income, while simultaneously creating new employment opportunities and generating new products to the market.

With the growth of Asian populations and economies, pig production in Asia is also increasing rapidly. Nevertheless, pig production in Asia still faces challenges in terms of availability of resources, low biological efficiency, environmental protection, and presence of diseases. Effects of pig production on economic growth and environmental sustainability should be considered carefully when developing regional and national plans.

The global pig production looks to Asia. Within Asia, all eyes are on China. China is the largest producer and consumer of pork in the world, accounting for about half of the world's pig production and pork consumption. Therefore, this paper aims to summarize current status and challenges of pig production in Asia based on the pig production situation in China. In addition, available practical approaches and future research foci are also proposed for sustainable development of pig production in the region.

## OVERVIEW OF THE PIG PRODUCTION IN ASIA

### Pig production

**Figure 1:**
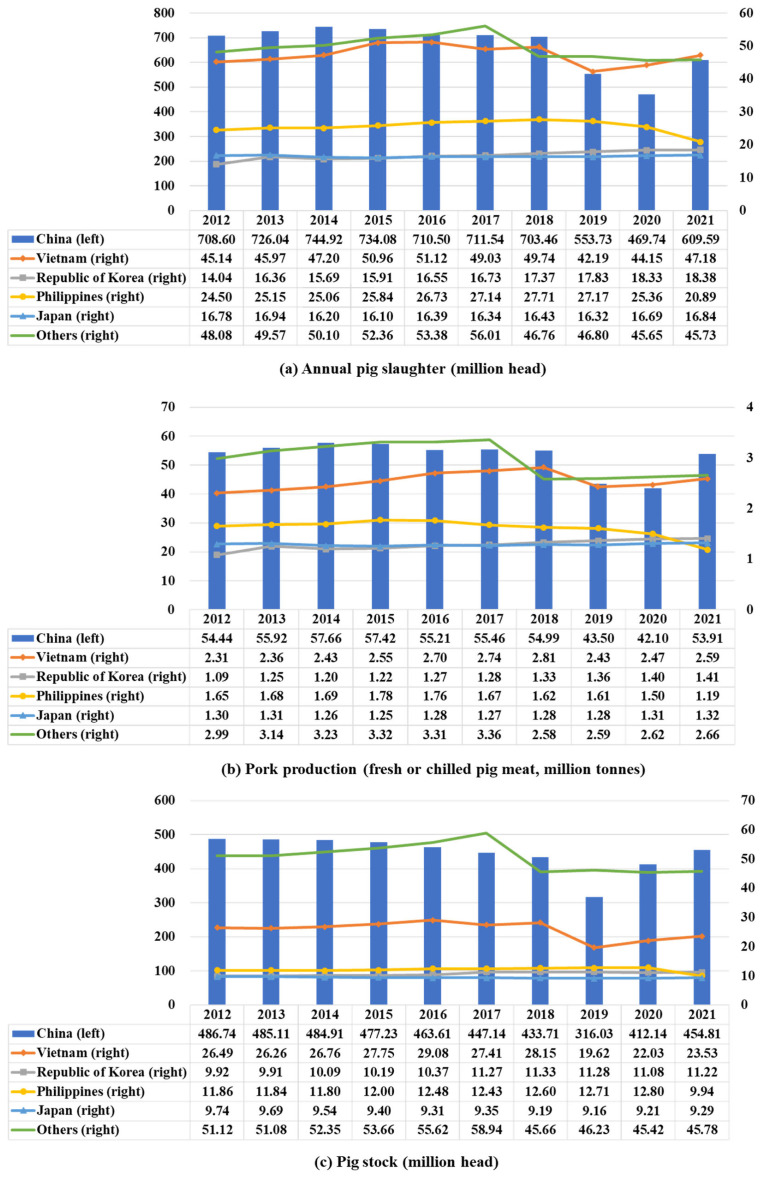
Changes in annual pig slaughter (a), pork production (b) and pig stock (c) in Asia over the last 10 years (data sourced from Food and Agriculture Organization [5]).

Pigs are one of the most widely raised animals in the world. In 2021, there were about 749.62 million pigs worldwide, of which Asia accounted for 74.0% [3]. From 2012 to 2018, pig slaughter in Asia exceeded 850 million head per year, and annual pork production exceeded 63 million tonnes (meat of pig with bone, fresh or chilled pig meat; Figure 1). However, due to the outbreak and spread of African swine fever (ASF) in Asia in 2018, both pig populations and pork production have plummeted [4], with annual pig slaughter falling to 704 million head and annual pork production to 52 million tonnes in 2019. In 2020, this situation got even worse as COVID-19 reduced pig slaughter 12% compared with 2019. After 2020, the pig production in Asia gradually recovered, with pig slaughter and pork production increasing 22.4% and 22.7%, respectively, in 2021 compared to 2020.

**Figure 2:**
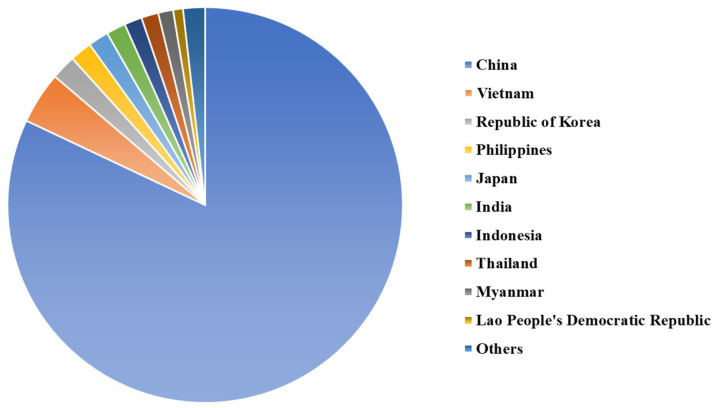
Pig stock in Asian countries in 2021 (data sourced from Food and Agriculture Organization [5]).

Subject to the influence of dietary habits, resource endowments, and religious and cultural factors, pig production in Asia exhibits distinct regional clustering characteristics, predominantly concentrated in China, Republic of Korea, and Japan in East Asia, as well as Vietnam, the Philippines, Thailand, and Indonesia in Southeast Asia (Figure 2). Unsurprisingly, China is the leading pig producer in Asia. The pig stock in China consistently accounts for 80% or more of the total pig population in Asia, reaching a peak of over 85%. By the end of 2021, China’s pig stock reached 454.8 million head (Figure 1), representing 82.0% of the pig population in Asia. Vietnam and Republic of Korea ranked second and third in pig stock after China, with their pig stock in 2021 amounting to 23.53 million and 11.22 million head (Figure 1), accounting for 4.2% and 2.0% of the pig population in Asia, respectively.

### Pork consumption

**Figure 3:**
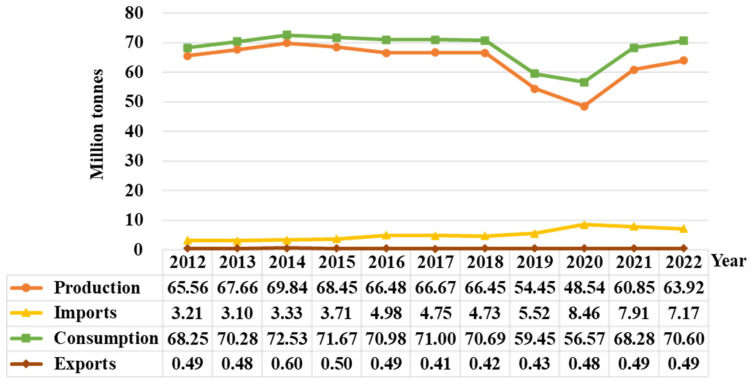
Changes in pork production, imports, consumption and exports (million tonnes, carcass weight equivalent) in Asia over the last 11 years (data sourced from Organisation for Economic Co-operation and Development [7]).

Pork has played a crucial role in diets for humans for thousands of years. With increasing demand for animal products, pork will continue as one of the primary sources of protein in Asia. Asian culture traditionally prefers pork consumption over other red meats. In 2022, pork consumption in Asia was about 70.6 million tonnes, accounting for 62.7% of global pork consumption [6]. From 2013 to 2018, pork consumption in Asia consistently exceeded 70 million tonnes (Figure 3). However, in 2019, the consumption of pork dropped significantly due to the impact of ASF. Consequently, there was a substantial surge in pork imports to meet the demands of Asian consumers during 2019–2020. Pork production in Asia is mainly for domestic consumption, with exports of less than 1 million tonnes annually (Figure 3).

China is world’s largest pig production and pork consumption country. In 2022, pork production in China was about 52 million tonnes, accounting for 44.15% of global pork production [6]. However, China still imported about 3.5 million tonnes of pork to supply domestic demand (Table 1). This is largely because Chinese culture traditionally prefers pork consumption over other red meats. The top five sources of imports, based on import volume, are Spain, Brazil, the United States, Denmark, and the Netherlands, which together accounted for 74.7% of China’s total pork imports in 2021 [8]. The pig production in Japan and Republic of Korea has encountered significant obstacles to pork production such as rising production costs, environmental restrictions, and infectious diseases. As a result, both countries have relied on increasing pork imports to supply their demand [9]. In 2022, imports pork in Japan and Republic of Korea accounted 51.0% and 33.3%, respectively, of total pork consumption (Table 1). Affected by the spread of ASF in Asia in 2018, pork human consumption per capita in China and Philippines in 2020 has declined rapidly, even below the level of pork human consumption per capita in 2000, while ASF had a less negative impact on pork consumption in Japan and Republic of Korea (Table 1). In addition, Republic of Korea, Vietnam, and China have the highest per capita pork consumption in Asia (Table 1).

### Pig production systems

**Figure 4:**
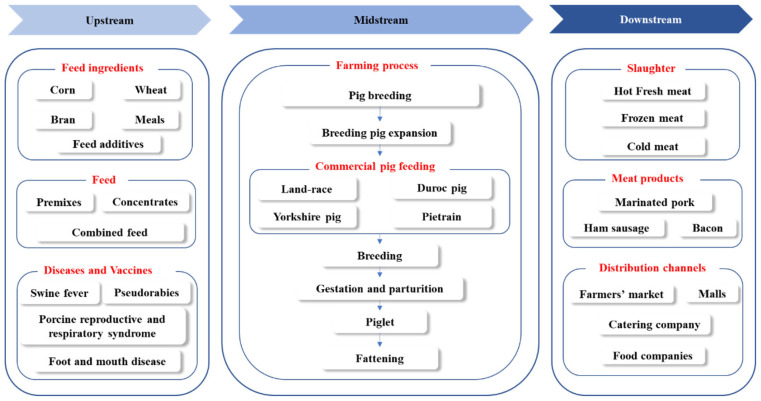
Value chain in pig production.

When considering the value chain in pig production, the upstream segment is composed primarily of pig feed manufacture, vaccines, and animal pharmaceuticals. The pig production occupies a middle position in the industry chain, while the downstream segment consists of pig slaughtering and meat processing, ultimately supplying the end consumer market with pork products (Figure 4).

Over the past three decades, pig production in many developing countries has undergone a rapid transformation from primarily small-scale pig farms to intensive production units. In developed countries, large-scale production systems have achieved high levels of production performance and become the predominant type of pig farming system. In developing countries, large- and medium-scale production systems have also been implemented. In Asia, however, the largest population of pigs still exists in traditional, small-scale production systems [10–14]. Small-scale farmers can be classified into two types: backyards and small-scale commercial farmers. Backyard farmers typically raise local pigs for breeding and/or fattening purposes [15]. Because small-scale farmers cannot compete with industrialized farms in terms of production costs and pig performance [16], future pig production systems in Asia will continue to shift towards intensive, large-scale, geographically concentrated, commercial, and specialized systems.

**Figure 5:**
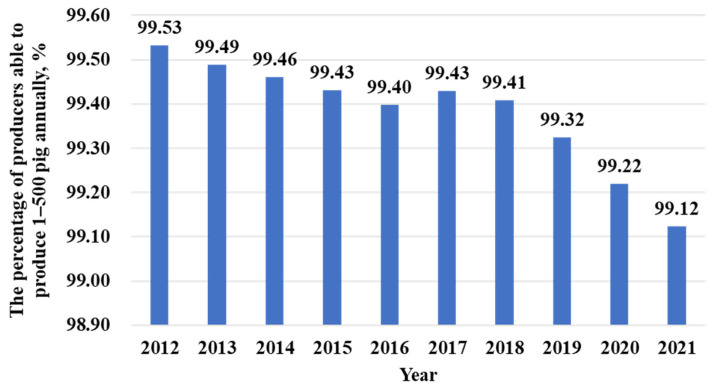
Changes in the proportion of small-scale farmers (produce 1 to 500 pig annually) in China from 2012–2021 (data sourced from Ministry of Agriculture and Rural Affairs of China [17]).

**Figure 6:**
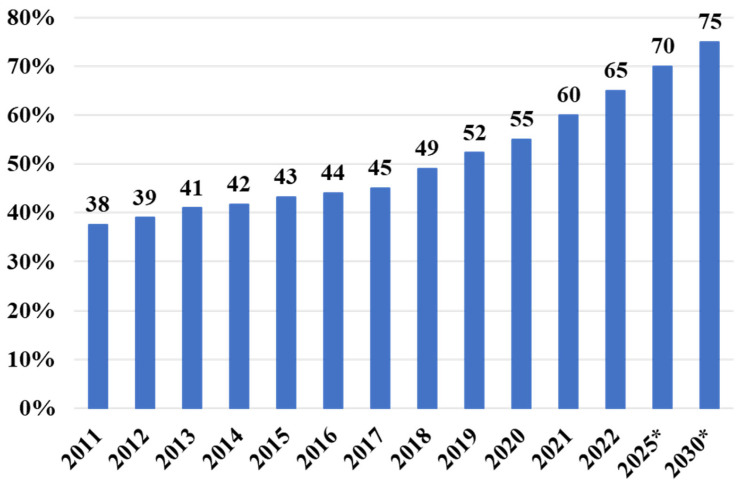
Changes in the proportion of pig production from large-scale pig farms (produce >500 pig annually) in China (* predicted values; data sourced from Ministry of Agriculture and Rural Affairs of China [17]).

In 2015, China’s pork sector encountered unprecedented restrictions due to the implementation of the new national environmental protection policy [13]. Some small-scale producers left the sector. The spread of ASF in 2018 further impacted the structure of the pig production sector, with many small-scale farmers leaving the industry (Figure 5). Pig farming in China is gradually shifting toward large scale operations. The proportion of pig production from large-scale pig farms (produce >500 pig annually) increased from 37.6% in 2011 to 65% in 2022, and is projected to reach over 75% by 2030 (Figure 6) [17].

**Figure 7:**
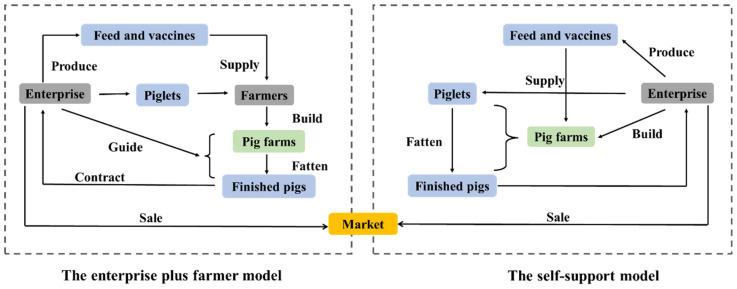
Schemes of two main organization models of large-scale modern pig production systems in China [13].

Large-scale modern pig production systems in China can be divided into two types: the enterprise plus farmer model and the self-support model (Figure 7) [13,18]. Currently, enterprise plus farmer model is the most common, which can rapidly promote the expansion of production scale. Under this contract-based model, farmers are only responsible for the fattening process, while the company or large-scale pig producer will provide piglets, feed, vaccines, and professional guidance. Once fattening is completed, the enterprise will collect market-ready pigs, undertake the marketing, and settle with the cooperative farmers based on the contract and market prices. In contrast, the self-support model is a system in which the enterprises are responsible for the entire process [13]. In the process of promoting development of large-scale production systems in China, both models have played a significant role in ensuring a sustainable pork supply and a stable market for pig production. Each model has respective advantages. The enterprise plus farmer model requires relatively less capital and is easily scalable. The self-support model has great advantages through its control hog quality, food safety, disease prevention and control, and production efficiency. In Vietnam, large-scale pig farms include two production models, namely intensive large-scale commercial farms with high levels of hygiene or collaboration with farms at a medium level of hygiene [11].

## CHALLENGES FACING PIG PRODUCTION

Although the pig production in Asia has made great progress in the past, it is currently confronted with numerous challenges. These challenges include inadequate land and feed resources, a substantial number of small-scale pig farms, escalating pressure to ensure environmental conservation, and devastating infectious diseases, as well as coping with high environmental temperatures and high humidity.

### Inadequate land and feed resources

Relative shortages of land and feed resources remain the major challenge for pork production in Asia. With the expansion of large-scale pig production, demand for land to site new production units continues to increase. Many countries have not yet included land for animal husbandry in their national spatial planning. In addition, urbanization will reduce agricultural land. Therefore, it is difficult to find suitable land for new large-scale pig farms.

Feed costs represent about 60% to 70% of the total cost of pig production. Asian countries are highly dependent on importation of animal feed [11,13,19]. In 2022, China imported 146.87 Mt of cereal and oilseed crops, comprised of 91.08 Mt soybean, 5.38 Mt barley, 10.14 Mt sorghum, 20.62 Mt maize, and 9.80 Mt wheat. More than 70% of these commodities were used for animal feed [8]. The current instability in global supply chains and international trade has increased the uncertainty and risk for development of the pig production in Asian countries. Prices of pigs and pork have not increased in the past three years but prices of the feed and raw materials have increased steadily. Soybean meal and corn are the primary raw materials for pig feed in China. In 2022, global soybean meal price hit a historic high, while corn price continued to run at high levels. These conditions inflated feed prices and total cost of pig production, which greatly exacerbated financial loss incurred in the pig production in Asia, especially China.

### Large proportion of small-scale pig farms

The definition of small-scale pig farms varies among countries. In China, pig farms that produce less than 500 head of pigs for slaughter annually are defined as small-scale. China currently has 20 million pig producers, however, only 180,000 of them are large-scale farms (produce >500 pig annually). In other words, more than 99% of the pig producers are small-scale farmers or part-time farmers (Figure 5) [20]. In the Philippines and Vietnam, a small farm has less than 20 pigs, while small farms in Cambodia and Laos have less than five pigs. The majority of pigs in these four countries are raised on small-scale farms [19]. In Thailand, farmers raising less than 50 pigs are considered small-scale farmers, who account for more than 90% of pig producers [10]. In fact, small-scale production systems still account for a large proportion of pig production in Asia. Due to the limited scale and inherent spontaneity of small-scale pig farms, government agents faced difficulties controlling costs and diseases of pig herds. Small-scale farmers tend to have higher feed wastage and lower production efficiency which contributes to significant environmental pressure compared with large-scale farms. Furthermore, for these small-scale pig farms, advanced technologies in breeding, nutrition and feeding, health care, housing, and management are not easy to apply in practice [21].

### Increased pressure on environmental protection

The rapid development of the pig production sector, coupled with inadequate management, incomplete regulations, and the spatial separation between crop and pork production systems, has led to a significant increase in environmental pollution caused by pig production [22–24]. Improper disposal of manure and slurry from pig farms results in pollution of water, air, and soil [25–28]. In addition, feed production accounts for most environmental impacts of pig production systems, primarily due to emissions of associated greenhouse gases and use of non-renewable energy and resources [29,30]. Therefore, greater effort is needed to preserve quality of the environment and promote the sustainable use of resources. Unfortunately, increases in costs of environmental protection compress, profit margins of pig production. The cost of waste disposal accounts for 4% to 19% of total costs in pig production systems. These expenses hinder implementation of waste disposal plans, particularly for small-sized farms [31]. On the other hand, large-scale pig farms have also faced criticism in studies, primarily due to improper handling and disposal of pig manure and its negative impact on the environment [28,32–34]. Intensive production systems have led to excessive accumulation of manure and slurry, as large-scale pig farms have no land-base for proper manure disposal, thus imposing additional pressure on the environment [35]. Therefore, the environmental protection pressure on pig production is growing. Regulators have implemented a series of environmental regulations to control pollution from pig production. In 2015, the Chinese government banned livestock production in some regions (called non-livestock production regions) to control surface water pollution near vulnerable water bodies, natural scenic places, and human residential areas [36]. As a result, by 2017, 0.26 million pig farms had been shut down, resulting in a reduction of the production of 4.6 million pigs [37]. In Vietnam, the Ministry of Agriculture and Rural Development has encouraged farmers to move livestock, such as pigs, out of residential areas [11]. In Thailand, the Agricultural Standard Committee established the “Standard for Good Agricultural Practices for Pig Farms”, which aimed to reduce the adverse impacts of intensive pig production both in epidemiological and environmental terms [38].

### Devastating of infectious diseases control

The outbreak of infectious diseases has seriously threatened pig production and has caused huge economic losses for producers. In some cases, these pathogens also impact human health. The major viral diseases that threaten pig production include: influenza, pseudorabies (Aujeszky’s disease), foot and mouth disease (FMD), porcine reproductive and respiratory syndrome (PRRS), classical swine fever and ASF. The major bacteria that threaten pig production include: *Salmonella*, *Escherichia coli*, and *Actinobacillus pleuropneumonia* (APP) [39]. The intensification and globalization of pig production has exacerbated emergence and spread of infectious diseases, partly due to the frequent movement of feed, pigs, and pork products at local, national, and international levels [40]. Indeed, infectious diseases of pigs are a significant factor that constrains pork production and trade in Asia [41,42]. As mentioned above, most pig producers in Asia are small-scale farmers who have a low level of knowledge and understanding regarding infectious diseases. Consequently, their capacity to implement disease prevention and control strategies is limited [43]. Over the last two decades, major infectious diseases such as FMD and PRRS have caused a direct impact on pig production in Asia countries, especially on farms with low biosecurity (small-scale pig farms) [11, 44–46]. In Asia, following the emergence of ASF virus in China in 2018, ASF has been reported in several other Asian countries, including Vietnam, Cambodia, North Korea, Laos, North Korea, Myanmar, Indonesia, Republic of Korea, India, and Thailand [47]. The spread of ASF has reduced pig populations and pork production, causing substantial economic losses to pig production in Asian countries [11, 14,48]. Insufficient understanding of the interaction of ASF virus with the host immune system hinders vaccine development [49]. The live attenuated vaccines of ASF can provide 100% protection against homologous strains, but there are still potential safety concerns [49]. The mRNA vaccine technology has made significant progress over the past two decades [50], which has recently proven effective in controlling COVID-19 [51]. However, mRNA vaccines are also expensive to manufacture and transport, and there may be potential unknown risks. Therefore, the development of ASF vaccine has a long way to go. Finally, growing demand for pork has led to intensification of production large-scale farms raising thousands of pigs in high-density conditions, can facilitate rapid transmission of pathogens. Consequently, threats from infection diseases will continue to pose a threat to healthy development of the pig production and food security in Asia in the foreseeable future.

### Other challenges

Other challenges such as unstable pork prices, low production efficiency, high temperatures and high humidity, and restricted use of antibiotics are significant threats to development of pig production. In China, rapid influx and swift withdrawal of capital have exacerbated volatility of pig production, leading to fluctuations in pork prices and forcing some vulnerable small farmers to exit production. This volatility undermines stability and sustainable development of pig production. Asian countries exhibit significant gaps in labor productivity, sow productive performance, and resource conversion efficiency when compared to developed countries. For instance, number of piglets weaned per sow per year is 25 in American and 30 in Denmark, while in China this number was only 17 [52]. Southeast Asian countries are situated in the equatorial climate zone, which is characterized by consistently high environmental temperatures and high humidity. These climate conditions have a significant impact on quality of semen produced by boars. Gilts experience reproductive dysfunction when exposed to high temperatures during pro-oestrus, leading to issues such as merciless, anovulatory delayed estrus, and ovarian cysts during estrus [10,14]. Moreover, reduced feed intake of pigs is a major limiting factor in swine production under tropical and humid climatic conditions [53]. Antibiotics were used in pig feed as effective growth promoters. However, the abuse of antibiotics during livestock production can cause serious antibiotic residues in animals. The development of antibiotic resistance in pigs that can later through food chain enter humans and reduce effectiveness of antibiotics in humans [54]. The Chinese Ministry of Agriculture and Rural Affairs has officially banned the use of antibiotics as feed additives from July 1, 2020 [55]. Therefore, there is an urgent need to find safe and effective alternatives for pork producers to remain competitive.

## PROSPECTS IN PIG PRODUCTION

In the foreseeable future, pork will continue to be the predominant meat consumed in the Asian region. Development of the pig production in Asia is closely related to issues of environment, economic benefits, and social sustainability. Therefore, strategies must address how to increase economic contributions of the livestock production and how to develop environmentally and resource-friendly, sustainable pig production systems.

### Breeding

Animal breeding can make an increasingly important contribution to sustainable food security [56]. Some strategies can be used to improve the breeding of pigs. Pig performance can be improved by developing non-contact intelligent measurement devices and utilizing image-based phenotyping technologies to upgrade existing genomic breeding technologies. These technologies most be implemented in core populations, breeding populations and commercial pig production systems. Accelerating cultivation of new genetic line with improved characteristics, such as fast-growth, high reproductive performance, or stress-resistant breeds will enhance pig production systems. In addition, establishing boar stations with high biosecurity and high-quality semen that provide high-quality genetics to small and medium-scale pig farms will advance a large proportion of the current pig production. Genetic diversity supports livestock adaptation to their environment. Different native breeds of pigs have specific distribution areas that provide a large natural gene pool. These native pig breeds are more highly adapted to their specific environmental conditions and feeding resources [57]. Protecting these genetic resources and more fully incorporating them into moder production systems will advance adaptation of pig production to the environmental conditions present in Asia [19].

### Feed ingredients

Improving feed utilization efficiency is an important approach to overcome the relative shortage of feed resources. One strategy is to feed pigs a diet composed of locally-available feed resources, such as agricultural by-products, without compromising their growth performance and meat quality [58–61]. Accurately assessing the nutritional value of these local ingredients and developing cost-effective processing technologies are control to the utilization of local feed resources. Another strategy is to promote precision formulation technology [62]. Development of precision nutrition in pig production relies on precise evaluation of nutritional value of feed ingredients and accurate determination of nutrient requirements of pigs under different conditions [62]. With implementation of smart devices in pig production, precision nutrition technology may be possible in the near future. In addition, development of low-protein diets supplemented with crystalline amino acids can improve efficiency of nitrogen utilization, reduce pressure on protein ingredient supplies, and provide environmental benefits [63,64].

### Structure of the industry

Although large-scale pig farms have been rapidly increasing in Asian countries in recent years, there is still a considerable gap compared to developed countries in Europe and the United States. In 2022, the proportion of pig production from large-scale pig farms (produce >500 pig annually) in China reached 65%, while in the European Union it is close to 90% and in the United States it is already over 95% [7]. Therefore, large-scale pig farms conglomerates will be more and more active and aggressive. More standardized and systemic pig farms will be established. Additionally, considering the large number of small-scale farmers in Asia, the enterprise plus farmer model and the production cooperatives model will be further promoted in the future [10,11,13]. On the other hand, natural and organic pig farming is being promoted. Small-scale pig farmers can integrate pig farming with a combination of other livestock, crops, vegetables, and fruit production as an integrated organic farming [65,66].

### Environment

In pig production system, feed production and waste management are the main aspects of environmental pollution. The environmental pressure caused by feed production can be alleviated by increasing the inclusion of agricultural by-products in diets, reducing the dietary protein level, and supplementing dietary enzymes to enhance digestion of feed nutrients [67]. In addition, development of anaerobic co-digestion of pig manure with agricultural wastes and promoting circular agricultural systems through integration of pig and crop production are all effective approaches to disposal of manure created by pigs [68]. In Japan, pig wastewater is mostly treated by activated sludge process to obtain clear water [69]. This is a good reference for farms that do not have enough cropland to use liquid matter. Antibiotics and inorganic minerals (Cu, Zn) added to pig feed have a direct impact on agricultural ecosystems and the environment [54]. In 2017, the Chinese Ministry of Agriculture and Rural Affairs lowered the limits of Cu and Zn use in feed additives by about 20% to 30% [55]. Therefore, development and use of alternatives to antibiotics and inorganic minerals, including plant-sourced essential oils, Chinese herbal extracts, organic acids, probiotics, antibacterial peptides, and metal oxide nanostructures, are needed to maintain sustainable development of pig production [54,70–73]. Finally, strategies such as strengthening supervision of emissions from livestock sector and establishing of emission reduction and carbon sequestration standards are vital for green development of agriculture in the future [74].

### Diseases

To ensure sustainable pig production in the long term, Asian countries need to strengthen prevention and control of pig diseases, especially ASF. Firstly, local authorities must establish and implement technical training and education programs to enhance knowledge of producers, particularly small-scale farmers, in the control of ASF [2]. Pig farm managers need to implement strong health measures for farms and farm workers to reduce the spread of various infectious diseases [75]. Further, government agencies must strengthen management of disease monitoring, quarantine supervision, and safe disposal of diseased pigs. Ultimately, research on vaccines and development of antiviral drugs represents more proactive strategies for disease control in currently infected countries. On the other hand, enhancing biosecurity, such as the decontamination and disinfection of transport trailers and other potentially contaminated fomites, supply of secure feed or feed additives, and regularly evaluate the surrounding environment of pig farms, is important to prevent the spread of swine infectious diseases by direct or indirect contact [76,77].

## CONCLUSION

Pork accounts for more than one-third of meat produced world-wide and is an important component of global food security, agricultural economies, and trade. Pig production in Asia is developing and expanding. However, due to relative shortage of feed resources, increased pressure on environmental protection and presence of devastating infectious diseases, costs of pig production are gradually increasing. With these challenges, exploitation of alternative feed resources, precise feeding, use of low-protein diets, development and use of vaccines and alternatives to antibiotics and inorganic minerals, and proper utilization of pig manure are all important topics and research areas. Small-scale farmers are the most vulnerable and affected segment, therefore, transformation of pig production to medium- and large-scale farms, together with enhanced standardization of production systems and biosecurity, will facilitate development of environmentally friendly, profitable, and sustainable systems of pig production in Asia into the future.

## Figures and Tables

**Table 1:** Pork production, trade and consumption (million tonnes, carcass weight equivalent) in selected Asian countries (2000–2022)

Item		China	Vietnam	Republic of Korea	Philippines	Japan
2000	Production	39.66	1.42	0.92	1.21	1.26
	Imports	0.07	<0.0001	0.19	0.03	0.93
	Consumption	39.67	1.40	0.98	1.24	2.18
	Exports	0.29	0.02	0.03	0.001	0.0002
	Human consumption per capita (retail weight, kg)	23.98	13.69	16.13	12.39	13.33
2005	Production	45.55	2.29	0.90	1.41	1.25
	Imports	0.04	0.0001	0.35	0.02	1.25
	Consumption	45.08	2.27	1.18	1.44	2.46
	Exports	0.65	0.02	0.02	0.0004	<0.0001
	Human consumption per capita (retail weight, kg)	26.42	21.13	18.86	12.98	14.95
2010	Production	51.38	3.04	1.11	1.64	1.29
	Imports	0.41	0.001	0.39	0.09	1.08
	Consumption	51.51	3.02	1.54	1.73	2.36
	Exports	0.28	0.01	0.0004	0.002	0.001
	Human consumption per capita (retail weight, kg)	29.35	26.80	24.31	14.34	14.35
2015	Production	56.45	3.49	1.22	1.78	1.27
	Imports	0.96	0.01	0.61	0.10	1.18
	Consumption	57.15	3.48	1.82	1.87	2.45
	Exports	0.25	0.02	0.004	0.003	0.002
	Human consumption per capita (retail weight, kg)	31.69	29.26	27.92	14.30	14.96
2020	Production	36.34	3.55	1.40	1.50	1.32
	Imports	5.28	0.23	0.56	0.09	1.36
	Consumption	41.52	3.76	1.98	1.59	2.71
	Exports	0.10	0.01	0.01	0.002	0.002
	Human consumption per capita (retail weight, kg)	22.50	30.16	30.18	11.33	16.68
2022	Production	52.00	3.79	1.37	1.10	1.30
	Imports	3.50	0.24	0.68	0.44	1.34
	Consumption	55.38	4.02	2.04	1.54	2.63
	Exports	0.13	0.01	0.007	0.002	0.003
	Human consumption per capita (retail weight, kg)	29.82	31.69	31.04	10.68	16.34

Data sourced from Organisation for Economic Co-operation and Development [7].
